# A Breathing Sonification System to Reduce Stress During the COVID-19 Pandemic

**DOI:** 10.3389/fpsyg.2021.623110

**Published:** 2021-04-12

**Authors:** Bavo Van Kerrebroeck, Pieter-Jan Maes

**Affiliations:** Department of Art History, Musicology and Theatre Studies, Institute for Psychoacoustics and Electronic Music (IPEM), Ghent University, Ghent, Belgium

**Keywords:** sonification, stress, synchronization, health and well-being, COVID-19

## Abstract

Since sound and music are powerful forces and drivers of human behavior and physiology, we propose the use of sonification to activate healthy breathing patterns in participants to induce relaxation. Sonification is often used in the context of biofeedback as it can represent an informational, non-invasive and real-time stimulus to monitor, motivate or modify human behavior. The first goal of this study is the proposal and evaluation of a distance-based biofeedback system using a tempo- and phase-aligned sonification strategy to adapt breathing patterns and induce states of relaxation. A second goal is the evaluation of several sonification stimuli on 18 participants that were recruited online and of which we analyzed psychometric and behavioral data using, respectively questionnaires and respiration rate and ratio. Sonification stimuli consisted of filtered noise mimicking a breathing sound, nature environmental sounds and a musical phrase. Preliminary results indicated the nature stimulus as most pleasant and as leading to the most prominent decrease of respiration rate. The noise sonification had the most beneficial effect on respiration ratio. While further research is needed to generalize these findings, this study and its methodological underpinnings suggest the potential of the proposed biofeedback system to perform ecologically valid experiments at participants' homes during the COVID-19 pandemic.

## Introduction

The COVID-19 pandemic has impacted mental health globally (Torales et al., 2020). Studies of previous pandemics show that during times of a pandemic, people exhibit fear and anxiety-related distress responses (Taylor, 2019). Although the consequences of the recent pandemic are yet to be understood, data has indicated widespread emotional distress leading to the definition of the COVID stress syndrome and stress scales (Taylor et al., 2020). While one area of research deals with the understanding of such consequences, another lies in the analysis of proper means to mitigate them. Research has already stressed the importance of interventions that can be delivered under pandemic conditions to reduce mental health issues and boost well-being (Holmes et al., 2020).

One avenue toward improved health and well-being is through music. Music can have physiological effects independent from individual preferences (Bernardi et al., 2009) and has shown benefits in therapeutic interventions. Specifically, music has shown its potential to reduce stress and anxiety in patients under hospital care through its facility to modulate arousal levels, regulate moods and by distracting patients from the experience of pain (Nilsson, 2009; Hunter and Schellenberg, 2010). Such music therapy has shown significant benefits as a support intervention to reduce stress and improve well-being in clinical staff working with COVID-19 patients (Giordano et al., 2020). Other musical activities such as in education have moved online and toward remote learning methods during the COVID-19 pandemic (Biasutti et al., 2021). Given the demonstrated flexibility of musical practitioners to adapt to these changes (Schiavio et al., 2021), distance-based musical interventions and methods for well-being and education could compensate for some of lost interpersonal interactions as well as create new ones (Philippe et al., 2020).

Another way of reducing stress and anxiety is through the practice of breath regulation (Harris et al., 1976; Clark and Hirschman, 1990). Breath pattern training can have a positive impact on patients with chronic obstructive pulmonary disease (Estève et al., 1996), a condition that increases the risk of developing a severe COVID-19 disease (Zhao et al., 2020). A goal of breath regulation is the modulation of (para)sympathetic activity, the so-called rest-or-digest and fight-or-flight response, resulting in increased relaxation or awareness and energy (van Dixhoorn, 1998). Breathing has a strong influence on heart rate variability and has been described as an interface for voluntary control of the autonomic nervous system (Jerath et al., 2006; Kox et al., 2014). This coupling between breathing and hearth rate is the core principle behind the technique of resonance breathing in which people regularly exercise the slowing of their respiratory rate to a resonance frequency of around 63 beats per minute, causing high oscillations in heart rate (Lehrer et al., 2000). Other research has shown the benefits of modulating the retention time (Kox et al., 2014; Jafari et al., 2016) or the inhale/exhale duration to a ratio of around 1:2 (Adhana et al., 2013; Van Diest et al., 2014).

In practice, breath regulation comes in two forms: through conscious control or by stimulus entrainment. When breath is guided by an external stimulus, it is often through some form of biofeedback (Lehrer et al., 2000). These biofeedback systems differ by the type of stimulus (Bergstrom et al., 2014), whether users are instructed or not (Moraveji et al., 2011), whether some behavioral or physiological measures serve as input to the system (Herath et al., 2018), the type of sensors (Ayoola et al., 2018), the data processing and representations (Feijs et al., 2010) or whether the biofeedback is individually presented or in group (Moran et al., 2016). While a lot of research has been done, many, if not all, of these systems lack a close and fluent adaptation to the user. While they often incorporate some form of tempo-alignment, they fail to take the breathing phase into account. Another shortcoming with instructed breathing is the fact that users have reported hyperventilation, which can influence their state of relaxation (Van Diest et al., 2014).

An effective way of providing biofeedback is through the use of sonification. Sonification is defined as “the transformation of data relations into perceived relations in an acoustic signal for the purposes of facilitating communication or interpretation” (Kramer et al., 2010). Although sonification emphasizes the conversion of data relations into sound, music can also be used as a tool for sonification (Bergstrom et al., 2014). Importantly, biofeedback can then leverage the reinforcing and rewarding aspects of music (Maes et al., 2016). Other studies have shown how musical stimuli can help to control breathing patterns (Fried, 1990; Harris et al., 2014; Reza Namazi, 2017). Leveraging the rewarding aspects of music and the informationally transparent features of sonification can thus help to both motivate, monitor and modify physiological and physical processes (Maes et al., 2016). However, this requires an analysis of causes and effects of biofeedback systems in order to formulate appropriate alignment strategies leading to the proper modification of behavior and physiology (Moens and Leman, 2015; Van Dyck et al., 2017). This paper could support such an analysis by proposing a distance-based experimental protocol and biofeedback system that uses sonification to modify breathing patterns and induce states of relaxation. The aim of this study is the validation of this protocol and biofeedback system and evaluation of three auditory stimuli in modulating respiration rate, ratio, relaxation and perceived pleasantness.

## Methods

### Participants

Eight females and 11 males were recruited using an online banner in the public Facebook group of the Institute of Psychoacoustics and Electronic Music (IPEM). One male was excluded due to a misunderstanding of the experimental instruction. There were no participants that reported any respiratory disorders, 10 participants had musical training (Mean = 11.7 years, SD = 10.3) and 15 participants performed physical activity of which 7 did this more than three times a week. No exclusion criteria were applied, participants were not compensated for their time and average age of participants was 34 years (SD = 8.61).

### Procedure

The study protocol was reviewed and approved by the ethical commission of the Ghent University. The experiment involved a within-subjects, repeated-measures design including three conditions of a breathing exercise in which different audio stimuli were tested.

Participants performed audio-guided breathing exercises of 8 min while indicating their breath cycles using arrow keys on their computer keyboards. The goal of each breathing exercise was to decrease the respiration rate to an optimal resonance frequency of ~6 beats per minute (Lehrer et al., 2000) and to increase the respiration ratio toward a healthier 1:2 inhale/exhale ratio (Adhana et al., 2013; Van Diest et al., 2014).

Each participant performed three trials of every condition and a baseline divided over 6 days which they were free to spread over a maximum of 2 weeks. Trial order was randomized between participants and each participant performed three trials on day 1, one trial on day 2–4, three trials on day 5 and the baseline trial on day 6. The baseline condition had no sound and was performed at the end of the experiment to incorporate learning effects and assure participants were used to breathing while indicating breathing onsets.

Experimental trials were conducted individually by participants at home for which they downloaded an application beforehand. At the start of the experiment, they met with the experimenter through a video call to verify sound playback, assure the use of headphones and receive instructions. During this first session, the experimenter stressed the importance of performing conditions in a comfortable position at around the same time in uniform contexts (same lighting, room, chair). The participant's task during each trial was to breath naturally and comfortably and indicate breath inhalation and exhalation using the left and right arrow keys on their keyboard. All participants were asked to set the volume to comfortable levels while excluding background sounds. They sat in front of their computer while looking at the application that showed an indication when they pressed the arrow keys on their keyboard.

**Figure 1:**
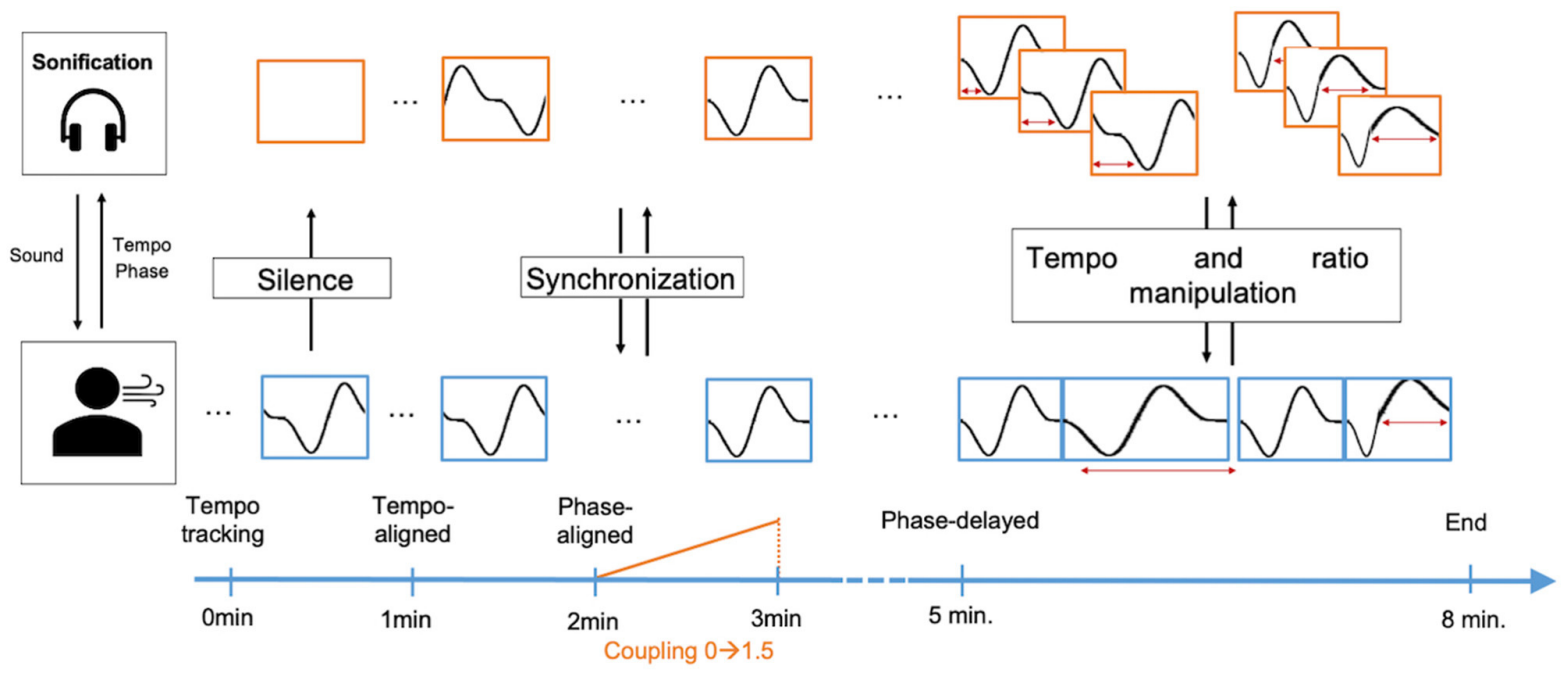
Biofeedback alignment strategy of breathing tempo and breathing phase.

Each trial lasted 8 min, consisting consecutively of 1 min of silence, followed by 7 min of an audio-stimulus. The stimulus was tempo-aligned to the participant's breathing cycles for 1 min, phase- and tempo-aligned for 3 min and then phase-delayed for 3 min (see Stimuli and Figure 1 for more details).

Participants were asked to fill in a questionnaire before and after each trial that evaluated their relaxation state, hyperventilation, and pleasantness of the stimulus. The experiment concluded with a questionnaire probing for COVID-19 related stress levels and appreciation of the breathing exercises.

### Stimuli

Each condition had a different stimulus that was modified using the same alignment strategy. The first condition, “noise” (No), consisted of amplitude and low-pass filter frequency modulated pink noise mimicking a human breath sound. The breathing sound was designed for a breathing cycle with an inhale/exhale ratio of 1:2 using smooth ramps toward the in- or exhalation onset. The second condition, “nature” (Na), aimed at providing a relaxing, ambient and acoustically immersive environment. It emulated a natural environment with wind-blown leaves, windchimes, soft rain and bird song sound samples and binaural playback of which the wind-blown leaves sound sample was spatially contracted and expanded based on the breath cycle phase. Specifically, using an ambisonics plugin (see Materials), eight duplications of the wind-blown leaves sound sample were placed at equidistant positions on a circle whose radius fluctuated with the breathing phase and was placed at anterior position of the listener. These samples were not time-aligned, were independently frequency filtered and amplitude modulated to prevent phase-cancellations and assure sounds differed enough to allow the spatial effect. The remaining sound samples were set at fixed, asymmetrical spatial positions around the listener. The third condition, “music” (M), played the stimulus from the noise condition on top of a decreasing arpeggio and 2-5-1 chord progression of a marimba-like sound, timed and played for an exhalation duration twice the length of inhalation. The noise sound was included to assure a continuous sound throughout the breathing cycle and because it created a sea-like sound in an ambient musical piece. When participants would indicate the start of an inhalation before the end of the phrase, the arpeggio and progression would quickly fade out. The rationale behind this design was that people would be spontaneously stimulated to wait for the harmonic resolution at the end of the phrase, and accordingly, to perform an optimal inhale/exhale ratio.

Tempo and phase-alignment between sonification and breathing as indicated by keypress onsets was done using an implementation of the Kuramoto model (Acebrón et al., 2005). The Kuramoto model is a mathematical model that models synchronization for coupled oscillators. It takes frequency, phase and a coupling constant of oscillators as inputs, and outputs a frequency for each oscillator that moves them toward synchronization. In our application, tempo was calculated by taking the median of the last five inter-onset-intervals of the participant's keypresses. Phase was set to 0 and 240 degrees at keypresses indicating, respectively the in- and exhalation. This ratio was chosen to ensure a fluent adaptation when the participant's respiration ratio approached the ideal 1:2 ratio. Between keypresses, phase was linearly interpolated based on the current breathing tempo. Phase-delay in the last 3 min of each session was varied between 0 and 50 degrees, based on the amount of synchronization between participant's breathing and the sonification stimulus measured using the mean vector length between the two phase vectors. We used this model as it has proven to be an effective means to adapt music to human behavior (Moens et al., 2010) and because the model allows a fluent adaptation of the stimuli's tempo to the respiration rate. An overview of the different phases in the alignment strategy is given in Figure 1.

### Materials

The biofeedback system was developed using the Max MSP framework,^1^ for the MacOS and Windows operating systems and distributed using a Github respository.^2^ Samples for the “nature” conditions were downloaded from the Freesound database (see Github for more details). Condition “noise” and condition “music” used stereo recordings. Ambisonics for binaural playback was used for condition “nature” and was created using the ICST ambisonic spatialization tools with a standard head-related transfer function and without head-tracking (Schacher and Kocher, 2006). Time stamps of arrow key presses on participants' keyboards were recorded locally in.txt files, which were sent to the experimenter after conclusion of the experiment. Behavioral data consisted out of the recorded timestamps of these keyboard presses during the trials and the baseline.

Psychometric data consisted out of measures of relaxation captured using 12 questions from the Smith Relaxation State Inventory Quick Test (SRSIqt) (Smith, 2001). We report four affective states from the questionnaire (basic relaxation, quiet focus, transcendence, positive emotion) and two stress dimensions (somatic and cognitive stress). Participants filled in the Nijmegen hyperventilation questionnaire (Van Doorn et al., 1982) to confirm natural breathing. A post-trial questionnaire asked participants how pleasant the sounds were (1 = not at all, 3–4 = moderately, 6 = a lot). A follow-up questionnaire asked for COVID-19 related stress effects, whether they would like to continue doing breathing exercises, when they would do them, and whether they would prefer them with or without music. Stress effects were measured using a rating scale with four questions developed for this study asking about participants' feeling of stress and social isolation in general and during the pandemic (two factors with Cronbach alphas >0.8 and ratings between 1 = not at all, 3–4 = average, 6 = a lot).

### Analysis

Two-way ANOVAs, with position (pre/post) and condition (No/Na/M/B) as within-subject factors together with a Kruskal-Wallis test on the follow-up questionnaire. Bonferonni correction were applied for multiple follow-up comparisons of significant interactions. Êta-squared effect sizes are reported below as well.

Respiration rate and ratio timeseries were calculated by taking the median Inter-Onset-Time (IOT) interval in a 6-keypress sliding window and timeseries were interpolated and resampled at 0.1 Hz (48 samples for each 8-min trial). IOTs and ratios that exceeded 5 times the standard deviation of the previous window were treated as erroneous presses and discarded ([Mean, SD] of the percentage of trial skipped: rates = [1.13, 1.92], ratios = [1.04, 1.69]).

As we were interested in evaluating the effects of the stimuli in decreasing the respiration rate and increasing the respiration ratio over time, we modeled timeseries with linear-mixed effects models using the LME4 package (Bates et al., 2014). Timeseries were log-transformed and modeled using a fourth-order orthogonal polynomial with a fixed effect of the condition on linear and all time terms for respectively, rate and ratio. Random effects consisted of participants and participants-by-condition-by-day on all time terms. Statistical significance for individual parameters was estimated using the normal approximation.

## Results

One participant did not perform day 6 of the experimental procedure. Two participants performed the wrong condition once, two other participants missed one trial on day 5. In total, 18 participants performed 175 trials.

Regarding the psychometric data, six participants filled in fewer questionnaires than prescribed by the protocol. One participant did not fill in the follow-up questionnaire. Two participants answered the pre- and post-trial questionnaires on day 5 only once, two other participants made the same mistake but on day 1, the remaining participants had 5 missing pre- or post-trial questionnaires on days 2, 3, or 4. In total, questionnaire data was received for 162 of the 175 trials.

### Psychometric Data

**Figure 2:**
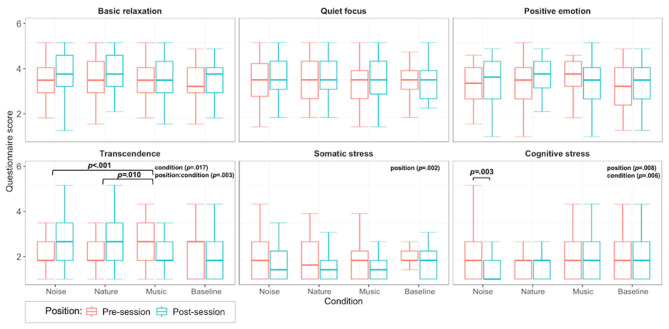
Boxplot of relaxation questionnaire data [subcategories taken from Smith (2001)].

Hyperventilation measures from the Nijmegen questionnaire assured a natural behavior of participants during each trial (mean = 5.52, SD = 4.28). Concerning stress and relaxation, we compared the six considered subcomponents of the SRSIqt (Smith, 2001) before and after each trial for all days (see Figure 2). Pre- and post-trial scores showed a significant effect for the relaxation component “transcendence” [*F*_(1, 301)_ = 5.756, *p* = 0.017, η^2^ = 0.018, 15 outliers removed] with a significant interaction effect between condition and pre/post trial scores [*F*_(1, 301)_ = 4.703, *p* = 0.003, η^2^ = 0.044]. *Post-hoc* tests showed significant increases for “transcendence” in post-trial questionnaires for the noise and nature conditions and decreased scores for the music condition ([pre-post, No-M]: *p* < 0.001, [pre-post, Na–M]: *p* = 0.010). There were significant effects for pre/post trial scores for the components “somatic stress” [*F*_(1, 306)_ = 9.428, *p* = 0.002, η^2^ = 0.03, 10 outliers removed] and “cognitive stress” [*F*_(1, 298)_ = 7.091, *p* = 0.008, η^2^ = 0.022, 18 outliers removed) with decreased scores across conditions. Finally, there was a significant effect across conditions on the “cognitive stress” component [*F*_(3, 298)_ = 4.307, *p* = 0.006, η^2^ = 0.040) with *post-hoc* tests indicating a significant decrease for the noise condition (*p* < 0.003).

Stimuli from the different conditions received the following average scores on perceived pleasantness ([Mean, SD]: B = [2.24, 1.30]; M = [3.43, 1.17]; No = [4.16, 1.36]; Na = [4.88, 0.89]). A Kruskal-Wallis test showed significant differences between scores [$\chi_{\left(3,162\right)}^{2}$ = 49.307, *p* < 0.001]. *Post-hoc* pairwise Wilcoxon Rank Sum tests indicated significant differences between all stimuli [*p*(No-Na) = 0.014, *p*(No-M) = 0.005, *p*(No-B) < 0.001, *p*(Na-M) < 0.001, *p*(Na-B) < 0.001, *p*(M-B) = 0.004).

Finally, in the follow-up questionnaire, participants did not indicate significantly higher stress levels during the COVID-19 pandemic as before (*p* = 0.29) yet did show a significant increase in terms of feeling of social isolation (*p* = 0.029). Thirteen out of 17 participants indicated they would continue doing the breathing exercises after the experiment, of which ten answered to prefer them with as compared to without music. A majority would do them at stressful or lonely moments and despite the COVID-19 pandemic, two participants indicated they would do them because of the pandemic.

### Behavioral Data

Respiration rate timeseries were analyzed for all participants for 173 trials. Two trials with sudden respiration rate peaks of 18.64 and 26.70 beats per minute were removed because of outliers in the timeseries and problems with model fitting (residual outliers).

**Figure 3:**
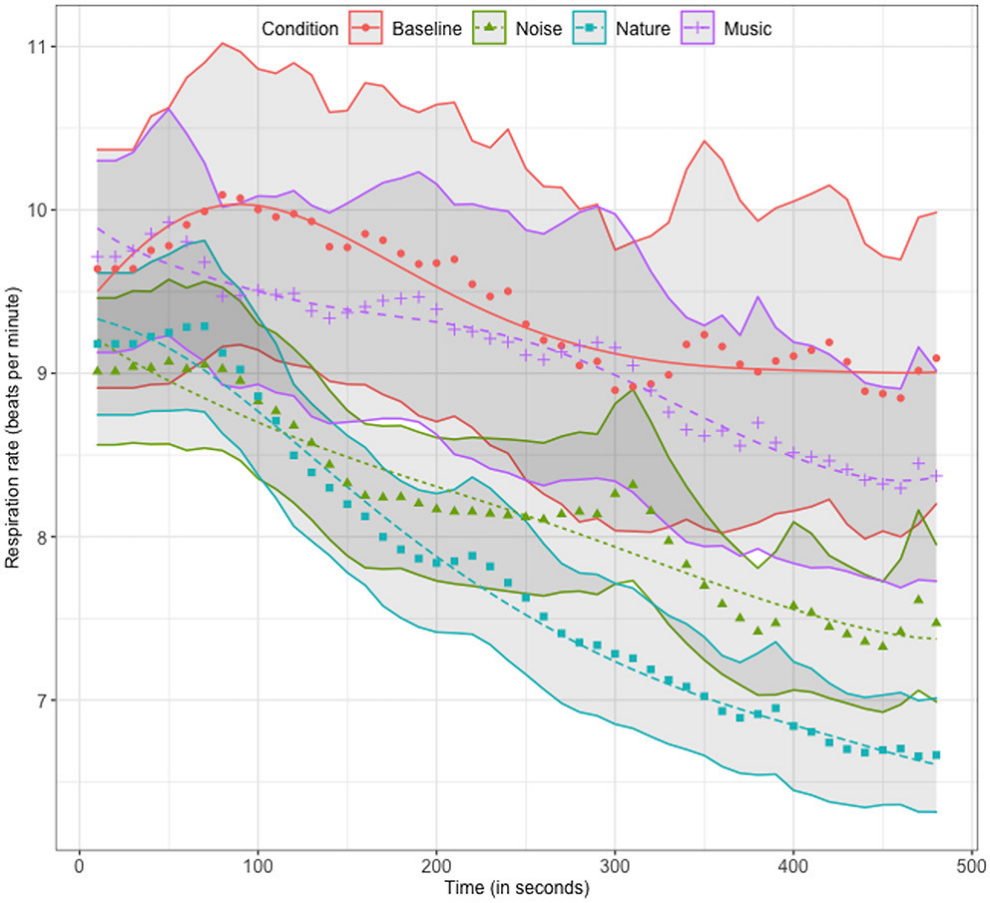
Mean, standard error and model fitted curves for the respiration rate timeseries for all conditions.

Mean respiration rate curves and model fits are shown in Figure 3. There were small significant effects of the noise and nature conditions on the intercepts ([Estimate, SE, *p*]: No = [−0.193, 0.099, 0.052]; Na = [−0.205, 0.099, 0.039]), indicating overall higher odds on a lower respiration rate at the start of a trial. Significant effects were found for these conditions on the linear slope time term indicating an overall significant decrease in respiration rate for the trial with as compared to without sounds ([Estimate, SE, *p*]: No = [−0.341, 0.155, 0.029]; Na = [−0.460, 0.155, 0.003]).

Respiration ratio timeseries were analyzed for 169 trials. Six trials with large respiration ratio fluctuations were removed because of outliers in the timeseries and problems with model fitting (residual outliers).

**Figure 4:**
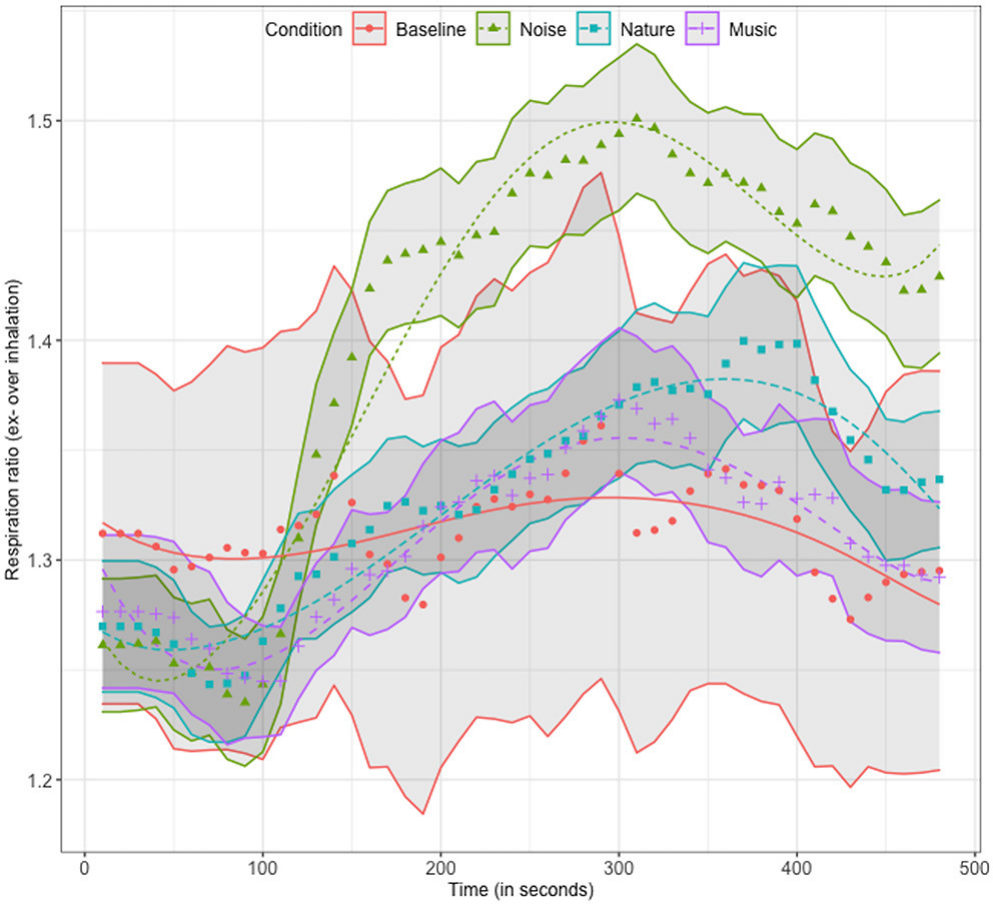
Mean, standard error and model fitted curves for the respiration ratio timeseries for all conditions.

Mean respiration ratio curves and model fits are shown in Figure 4. Large standard error bands are explained by the individual differences captured by the random effects in the model and one participant with a stable high ratio for the baseline condition ([Mean, SD] = [2.24, 0.17]). There were significant effects of condition on most time terms for the noise condition ([Estimate, SE, *p*]: linear = [0.363, 0.102, 0.0004]; quadratic = [−0.222, 0.074, 0.003]; quartic = [0.096, 0.045, 0.035]). As illustrated in Figure 4, the effects indicate an increasing ratio over time with stable plateaus at the beginning and end of the trial for the noise condition.

## Discussion

The aim of the present study was to assess the effects of a newly developed breathing sonification system on stress levels in persons during the COVID-19 pandemic. Our starting point thereby was the idea that stress can be reduced, and relaxation increased by modulating breathing patterns demonstrated by earlier research. We proposed the use of music and sound to effectuate spontaneous breathing pattern adaptation toward these goal states rooted in their combined arousal (Nilsson, 2009; Hunter and Schellenberg, 2010; Bergstrom et al., 2014) and (spontaneous) synchronization effects (Maes et al., 2019). The present study applied these principles of music and sound into a sonification system for spontaneous breathing adaptation. We specifically tested the effect of three types of sonification: (1) a noise sonification simulating a natural breathing sound; (2) a nature environmental sonification with ambient and spatially expanding/contracting sounds; (3) a musical sonification with tension and resolution in chord progressions and arpeggios. We assessed effects on breathing behavior as well as on perceived pleasantness, relaxation and stress levels.

Concerning the results on breathing behavior, it was found that the nature sonification led to the most prominent decrease in respiration rate. Interestingly however, it was the noise sonification that had the most beneficial effect on the respiration ratio. This differentiated effect on breathing behavior, depending on the sonification type, is likely to be explained by the specific auditory quality of the sonification. Potentially, the nature sonification with an ambient atmosphere may prompt arousal mechanisms, while the noise sonification with human breath-like sounds may rather spur synchronization mechanisms. This explanation is supported by findings from earlier research that have indicated the positive physiological effects of being immersed in natural soundscapes following stress (Alvarsson et al., 2010; Ratcliffe et al., 2013; Medvedev et al., 2015) and the existing link between perceived pleasantness and arousal from listening to soundscapes (Hume and Ahtamad, 2013; Aletta et al., 2018). On the other hand, sensorimotor synchronization mechanisms might have facilitated the changing respiration ratio resulting from the noise sonification. Since this sonification mimicked human breathing, in addition to rhythmical entrainment (Bardy et al., 2015), participants might have “mirrored” these sounds as in the use of ecological breathing sounds (Murgia et al., 2016). Though the underlying mechanisms may not be clarified, the finding urges to consider a combination of both sonification types in future research to maximize breathing adaptation effects.

Next to the physical breathing measures, we collected psychometric data to assess subjectively perceived pleasantness, relaxation and stress levels. Participants reported, on average, higher feelings of social isolation during the COVID-19 pandemic, but no significant higher stress level. However, across the different sonification types, the intervention led to significant reductions in stress and increased relaxation. Looking at the specific effects of the sonification type, we found that both the noise and nature sonification led to significantly higher increase of the experienced “transcendence” dimension compared to the musical sonification. We found “cognitive stress” to be significantly lower using the noise sonification and the nature sonification as the most pleasant. Finally, a large portion of participants (13 out of 17) reported that they would be willing to continue using our breathing sonification system in the future. From that portion, 10 participants pointed out that they had a preference for using it with music demonstrating the added value of the intervention.

The main outcome of the present study is that it offers an effective distance-based protocol and biofeedback system to improve interventions for breathing adaptation and stress regulation. Such a protocol could find applications in music therapy as a distance-based intervention for relaxation or in music education as a remote learning method of behaviors. Results indicate that a combination of the noise nature sonification is the proper candidate for future research and application. Both types of sonification contribute positively to distinct aspects of experience and adaptation in physical breathing behavior. Further research with larger sample sizes from heterogeneous populations with different backgrounds in for example music or meditation, studied at home and in the lab could confirm the expectation that their combination will lead to optimal results as well as reveal discriminatory effects resulting from individual profiles.

To generalize these preliminary findings, further research should compare results with those from post-pandemic experiments performed both at participant's home and in the lab as well as investigate the influence of exposure conditions. While performing experiments at participants' home increased the validity of the results, it also increased variability. Further research could control for this variability by an in-depth characterization of exposure conditions such as ambient sounds, headphone type, lighting, and psychosomatic factors during the introductory session with the participant. Another complementary way would be to incorporate automated controls such as for example headphone screening tests (Woods et al., 2017; Milne et al., 2020), a gold standard (Nelson and Allen, 2019) and background level recordings (Murphy and King, 2016). In future research, we plan to use a portable, custom-made sensor system that we developed to automatically track breathing cycles which allows to free the cognitive resources required to manually indicate breathing onsets. These studies will explicitly target physiological measures that indicate stress levels, such as heart rate variability and skin conductance, providing a better view on effects realized by our intervention application. In addition, while we induced a spatial effect in this study on manually positioned sound sources using ambisonics and a standard head-related transfer function, a possible improvement could be to incorporate head-tracking and individualized head-related transfer functions as it has shown to enhance binaural playback (Katz and Parseihian, 2012; Hong et al., 2019).

The COVID-19 pandemic poses substantial challenges to empirical research, in particular the data collection aspect, given the requirement of self-isolation and social distancing. One of the important contributions of our breathing intervention application is that it allows to collect quantitative data in a reliable and accurate manner, while participants can stay at home. This allows not only to cope with COVID-19 regulations, but equally to collect data within the participants' familiar, real-life environment, making them feel more at ease as compared to a laboratory environment.

## Data Availability Statement

The datasets presented in this study can be found in https://github.com/ArtScienceLab/SonicBreathing.

## Ethics Statement

The studies involving human participants were reviewed and approved by Ghent University Ethics Committee. The participants provided their written informed consent to participate in this study.

## Author Contributions

BV designed the study, conducted experiments, analyzed data and drafted the manuscript. PM designed the study, revised and approved the manuscript. All authors contributed to the article and approved the submitted version.

## Conflict of Interest

The authors declare that the research was conducted in the absence of any commercial or financial relationships that could be construed as a potential conflict of interest.

## Footnotes

^1^https://cycling74.com/.

^2^The application can be downloaded here: https://github.com/ArtScienceLab/SonicBreathing.
